# Stabilization of Inverse Miniemulsions by Silyl-Protected Homopolymers

**DOI:** 10.3390/polym8080303

**Published:** 2016-08-12

**Authors:** Sarah Wald, Frederik R. Wurm, Katharina Landfester, Daniel Crespy

**Affiliations:** 1 Department of Physical Chemistry of Polymers, Max Planck Institute for Polymer Research, 55128 Mainz, Germany; wald@mpip-mainz.mpg.de (S.W.); wurm@mpip-mainz.mpg.de (F.R.W.); 2 Department of Materials Science and Engineering, School of Molecular Science and Engineering, Vidyasirimedhi Institute of Science and Technology (VISTEC), Rayong 21210, Thailand

**Keywords:** miniemulsion, nanocapsules, phase transfer, radical addition fragmentation transfer polymerization, surfactant

## Abstract

Inverse (water-in-oil) miniemulsions are an important method to encapsulate hydrophilic payloads such as oligonucleotides or peptides. However, the stabilization of inverse miniemulsions usually requires block copolymers that are difficult to synthesize and/or cannot be easily removed after transfer from a hydrophobic continuous phase to an aqueous continuous phase. We describe here a new strategy for the synthesis of a surfactant for inverse miniemulsions by radical addition–fragmentation chain transfer (RAFT) polymerization, which consists in a homopolymer with triisopropylsilyl protecting groups. The protecting groups ensure the efficient stabilization of the inverse (water-in-oil, w/o) miniemulsions. Nanocapsules can be formed and the protecting group can be subsequently cleaved for the re-dispersion of nanocapsules in an aqueous medium with a minimal amount of additional surfactant.

## 1. Introduction

Nanocapsules, core-shell nanoparticles with a liquid core, have found successful applications in controlled and targeted delivery of therapeutics [1,2,3] or as contrast agents [4]. Nanocapsules can be generated by different techniques such as the layer-by-layer self-assembly [5,6,7], the (nano)precipitation method [8], solvent evaporation [9,10,11], or miniemulsion polymerization [12]. The miniemulsion polymerization process is suitable for the synthesis of well-defined nanocapsules because of the high stability of the miniemulsion droplets generated. With this method, nanocapsules with a liquid core can be prepared in a single step [13] with high encapsulation efficiency [12,14,15,16,17]. Their properties can be controlled by the amount and type of surfactant and osmotic pressure agent, the uniformity and intensity of energy input used to create the dispersion, and the monomer polarity. Surfactants are usually needed during the process to decrease the interfacial tension between the two phases and to stabilize the resulting nanocapsules [12,18,19]. In miniemulsion, the droplets are not densely covered with the surfactant molecules [20]. Oil-soluble non-ionic surfactants [21,22,23] and/or amphiphilic block copolymers are usually employed as stabilizers with low hydrophilic/lipophilic balance in water-in-oil emulsions [20,24,25]. Most of these amphiphilic block copolymers possess a poly(ethylene glycol) (PEG)-based hydrophilic block and are distinct in the hydrophobic block as well as the length of the different blocks [15,26,27,28,29,30,31,32]. The PEG-based amphiphilic block copolymers can be synthesized by anionic ring opening polymerization, a polymerization technique with a living character [33,34], so that the surfactants have a narrow molecular weight distribution and a precise block length. Other amphiphilic block copolymers can be generated by controlled radical polymerization techniques. The reversible addition–fragmentation chain transfer (RAFT) polymerization technique used in this paper was reported by Rizzardo and coworkers [35]. The principle consists of introducing a thiocarbonyl thio compound acting as a reversible chain-transfer agent (CTA). RAFT polymerization can be used for a wide range of monomers with a large variety of polymerization conditions and solvents [36,37,38].

For biomedical applications, nanocarriers loaded with hydrophilic cargo synthesized in inverse miniemulsions have to be transferred in water. Therefore, two surfactants are needed during the process. The first one is an oil-soluble surfactant with a hydrophilic–lipophilic balance (HLB) value of 4–8 and stabilizes water droplets in the oil phase. The second surfactant is water-soluble, possesses a larger HLB value (8–18), and stabilizes the nanocapsules re-dispersed in water [25]. During the transfer of the nanocapsules into water, the oil-soluble surfactant remains at the surface of the nanocapsule. This fact can have detrimental effects on the nanocapsule functionality [15,39,40,41]. To overcome this problem, surfactants that can switch their amphiphilicity on demand were developed. Such surfactants possess functionalities that can be triggered by different stimuli [42]. Examples of pH-switchable surfactants include carboxylic [43], tertiary amine [44,45], or imidazole groups [46,47]. Müllen et al. [48] reported the synthesis of a PEG-based surfactant for inverse emulsions with a photocleavable group in one block. Before deprotection, the block copolymer is soluble in the oil phase and can stabilize the poly(l-lactide) (PLLA) nanoparticles in the oil phase. During the transfer into water, the protection groups can be cleaved by light and stabilize the nanoparticles in water.

Herein, we simplify the procedure by using a protected homopolymer. Triisopropylsilyl protected poly(acrylic acid) was used as surfactant in inverse miniemulsions. The surfactant properties of the polymer were first tested by stabilizing droplets of water and formamide in cyclohexane. The miniemulsion droplets were then used as nanoreactors [15] to form polyurea (PU) nanocapsules. The triisopropylsilyl protection group was cleaved during the transfer step of the nanocapsules from an organic dispersion into an aqueous dispersion in order to form a hydrophilic polymer. Because the hydrophobic block of amphiphilic stabilizers shields the chemistry of the nanocapsule surface, they have a strong influence on further grafting of functional biomolecules and on the protein corona. This problem of generally used amphiphilic polymers can be overcome by using a cleavable homopolymer as surfactant in miniemulsions.

## 2. Materials and Methods

### 2.1. Materials

2,2′-Azobis(2-methylpropionitrile) (AIBN; Acros Organics, Geel, Belgium, 98%) was recrystallized in MeOH before used. Triisopropylsilyl acrylate (TIPSA; CHEMOS GmbH, Regenstauf, Germany), 4-cyano-4-(phenylcarbonothioylthio)pentanoic acid (Sigma-Aldrich, St. Louis, MO, USA, 97%), sodium dodecyl sulfate (SDS; Alfa Aesar, Ward Hill, MA, USA, 99%), sodium chloride (Sigma-Aldrich, 99.5%, St. Louis, MO, USA), 1,4-diaminobutane (DAB; Fluka, 98%, Buchs, Switzerland), toluene-2,4-diisocyanate (TDI; Fluka, 99.9%, Buchs, Switzerland), and toluene (Sigma-Aldrich, anhydrous 99.8%, St. Louis, MO, USA) were used as received. The solvents tetrahydrofuran (THF), methanol (MeOH), and cyclohexane were of analytical grade. Formamide (Fluka, 99%, Buchs, Switzerland) was dried over 4 Å molecular sieves before used. Dichlormethane-*d_2_* (Roth, 99.5% atom%D, Karlsruhe, Germany), dimethylsulfoxide-*d_6_* (Roth, 99.8% atom%D, Karlsruhe, Germany), deuterium oxide-*d_2_* (Sigma-Aldrich, 99.9% atom%D, St. Louis, MO, USA) and cyclohexane-*d_12_* (Sigma-Aldrich, 99.6% atom%D, St. Louis, MO, USA) were used as received.

### 2.2. Analytical Tools

Size exclusion chromatography (SEC) carried out in THF was used to detect the molecular weights of the synthesized polymers and their molecular weight dispersity (Ɖ) with an Agilent PSS SECcurity. The concentration of the samples was 5 mg·mL^−1^. After being filtered through a 0.45 μm Teflon filter, the samples were injected. The elution rate through the three SDV columns (PSS) was 1 mL·min^−1^. The SDV columns with dimensions of 300 mm × 80 mm have a particle size of 10 μm and pore sizes of 106, 104, and 500 Å. For detection a UV (254 nm) S-3702 detector (Polymer Standard Service GmbH, Mainz, Germany) and a DRI shodex RI-101 detector (ECR) (Polymer Standard Service GmbH, Mainz, Germany) were utilized. The molecular weights were calculated by comparing with a polystyrene standard provided by the Polymer Standards Service GmbH (Mainz, Germany).

^1^H-NMR spectra were measured on a Bruker Avance 300 spectrometer (Bruker, Billerica, MA, USA) operating at 300.23 MHz Lamor frequency. In 0.5 mL CD_2_Cl_2_ 15 mg of the synthesized polymers was dissolved and the spectra were calibrated according to the chemical shift of 5.32 ppm. For the studies of deprotection of the TIPS group, the reaction solution was measured every hour. The spectra were calibrated according to the chemical shift of 2.5 ppm (DMSO-d_6_).

^13^C-NMR spectra were measured using a 700 MHz Bruker Avance III spectrometer (Bruker, Billerica, MA, USA). In 0.6 mL deuterated dichloromethane (CD_2_Cl_2_) 30 mg of the synthesized polymer was dissolved and the spectra were calibrated according to the chemical shift of 1.38 ppm.

Dynamic light scattering (DLS) was measured with a Nicomp 380 Submicron Particle Sizer (PSS-Nicomp) (Particle Sizing System, Port Richey, FL, USA) at a fixed scattering angle of 90° to detect the z-average hydrodynamic diameter of the nanocapsules. 10 μL of the emulsion was diluted in 1000 μL cyclohexane or distilled water.

For nanocapsule detection a JEOL 1400 transmission electron microscope (TEM) with a LaB6 cathode (JEOL GmbH, Eching, Germany) was used. The copper grid had been modified with a carbon film (200 mesh, Science Services, Munich, Germany), before the TEM specimen was prepared. Therefore, the nanocapsules were diluted in cyclohexane or water and drop-cast on a copper grid. After drying of the TEM grid at room temperature, it was inserted into a sample holder and transferred into the TEM. The TEM was operated at an acceleration voltage of 120 kV.

Scanning electron microscopy (SEM) was carried out on a Zeiss 1530 LEO Gemini microscope (Carl Zeiss, Oberkochen, Germany). The working distance was ~3 mm and the accelerating voltage 0.2 kV. The nanocapsules were diluted in cyclohexane or water, drop-cast onto silica wafers, and dried under ambient conditions.

10 μL of the nanocapsule dispersion was diluted with 3 mL cyclohexane or distilled water and placed on silica platelets (SEM) or on a carbon-coated grid (TEM).

Infrared (IR) spectroscopy was performed on a PerkinElmer Sprectrum BX FT-IR spectrometer (PerkinElmer, Shelton, CT, USA). The range of the wavelength was between 4000 cm^−1^ and 400 cm^−1^. For solid samples, 3 mg of the nanocapsules were mixed with KBr, pressed and subsequently measured.

The interfacial tensions were measured with a ring tensiometer DCAT 21 from DataPhysics (DataPhysics, Filderstadt, Germany). The obtained value of cyclohexane in water (γ = 48.7 mN·m^−1^ at 22 °C) was comparable to the value reported in the literature (γ = 50.2 mN·m^−1^ at 20 °C) [49].

### 2.3. Analytical Tools

TIPSA (5.50 mL, 21 mmol) and AIBN (10.46 mg, 0.06 mmol) were added to 4-cyano-4-(phenylcarbonothioylthio)pentanoic acid (55.6 mg, 0.2 mmol) in a dry Schlenk flask and dissolved in 5 mL dry toluene. After three freeze-pump thaw cycles, the mixture was stirred at 70 °C for three days under nitrogen. The polymer was precipitated into cold methanol and dried in vacuo. Yield = 77%.

^1^H-NMR (300 MHz, CD_2_Cl_2_, δ): 7.38 (t, 5H, Ar H), 2.71–1.42 (m, 10H, CHCH_2_, CH_2_CH_2_, CH_3_), 1.29 (h, *J* = 6.9, 4.9 Hz, 3H, 3*CH), 1.15–0.92 (m, 18H, 6*CHCH_3_); ^13^C-NMR (750 MHz, CD_2_Cl_2_, δ): 13.19 (C1–C6), 19.79 (C7–C9), 44.39 (C10–C16), 128.8 (C17–C20), 130.27 (C21,C22), 175.99 (C23,C24); IR (KBr): ν = 3416 (Br), 2954 (s), 2872 (s), 2724 (w), 2375 (w), 1719 (s, C=O), 1467 (s), 1396 (m), 1371 (m), 1336 (w), 1266 (s, C–O), 1185 (s, C–O), 1115 (m), 1069 (m, Si–O–C), 1017 (m), 1001 (m), 923 (m), 885 (s, Si–C), 738 (s), 685 (s), 571 (m), 512 (m), 460 cm^−1^ (m); *M_n_* (SEC) = 10,100 g·mol^−1^; *M*_w_/*M*_n_ (SEC) = 1.67.

### 2.4. Preparation of the Inverse Miniemulsions

The dispersed phase containing NaCl (7.56 mg) and DAB (25 μL) in formamide (375 mg) was added dropwise to a solution of PTIPSA (45 mg) in cyclohexane (3.75 g) at room temperature. After stirring at 1000 rpm for 1 h, the emulsion was subjected to ultrasonication under ice cooling with a Branson W450-D sonifier equipped with a ½ inch tip for 3 min in a pulse-pause regime of 30 s and 10 s. TDI (54 μL) dissolved in cyclohexane (1.25 g) was added drop-wise to the emulsion and stirred for 24 h at room temperature. The size and morphology of the nanocapsules size and morphology were analyzed by DLS (Particle Sizing System, Port Richey, FL, USA) and SEM/TEM (Carl Zeiss, Oberkochen, Germany and JEOL GmbH, Eching, Germany) measurements. The capsules dispersion was washed three times by centrifugation at 3000 rpm for 15 min at 22 °C in 2 mL Eppendorf tubes (Eppendorf, Hamburg, Germany) to remove unreacted monomers. For re-dispersion of the nanocapsules into water, the washed miniemulsion (1 g) was added dropwise into a 0.1 wt % SDS solution (5 g) and stirred for 24 h without cap. Afterwards, the aqueous dispersion was dialyzed against distilled water for 36 h (average pore size of the membrane 14,000 g/mol) and analyzed by DLS and TEM/SEM.

### 2.5. Deprotection of TIPSA in Solution

TIPSA (100 μL) was dissolved in DMSO-*d_6_* (1.45 mL) and D_2_O (50 μL). After the addition of TFA (116 μL or 11.6 μL), the mixture was stirred at 1000 rpm. Every hour a sample was measured by ^1^H-NMR. After 1 h, the deprotection was completed. Furthermore, deprotection was studied without the addition of TFA. After 1 h, 2 h, 1 day, 2 days, and 3 days a sample was measured by ^1^H-NMR spectroscopy. Partial deprotection could be determined after 2 days.

## 3. Results

A desirable polymer surfactant for inverse miniemulsions should stabilize water-in-oil miniemulsions and then stabilize the formed colloids when they are re-dispersed in water. Therefore, we introduced switchable groups in the side-chain of the polymers. The soluble polymer backbone consisted in the water-soluble poly(acrylic acid) that was connected to hydrolysis-labile protecting groups. When the side-chains of the polymer are not cleaved, the polymer is oil-soluble. Acrylic acid with trialkylsilyl protecting groups was used as monomer, triisopropylsilyl acrylate (TIPSA), because the TIPS-group is easy to cleave under rather mild basic and/or acidic hydrolysis reactions [50,51,52,53,54]. On the other hand, the hydrolysis kinetics of the TIPS groups are relatively slow in the presence of the aqueous dispersed phase of water-in-oil emulsions. Indeed, the isopropylsilyl protection group is 700,000 times more stable towards acid catalyzed hydrolysis than the trimethylsilyl protection group, because the three isopropyl substituents show a strong steric screening for the silicon and also to the atom to which silicon is connected [55,56,57,58].

**Figure 1:**
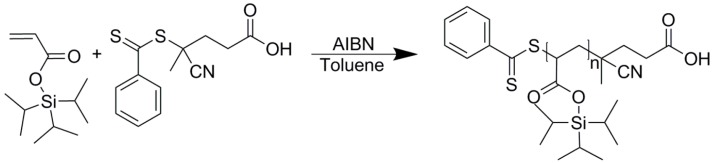
Synthesis of polymerized triisopropylsilyl acrylate (PTIPSA) by reversible addition–fragmentation chain transfer (RAFT) polymerization.

RAFT polymerization was used to prepare polymers with adjustable molecular weights that are suitable for the stabilization of the inverse miniemulsion droplets, as demonstrated by interfacial tension measurements. The polymers (PTIPSA) were synthesized as shown in Figure 1 with different molecular weights by changing the monomer: initiator ratio. Two polymers with molecular weights of 4600 g·mol^−1^ and 10,100 g·mol^−1^ and a molecular weight dispersity of *M*_w_/*M*_n_ = 1.19 and 1.67, respectively, were generated, characterized by ^1^H-NMR, ^13^C-NMR, FT-IR and SEC (Figures S1–S3) and used as stabilizers in the inverse miniemulsion process. The prepared molecular weights are typical for inverse miniemulsions and correspond to short polymer chains [59]. The polymer chains are sufficiently small to allow for rapidly reaching the adsorption equilibrium at the surface of the droplets, and are large enough to allow efficient steric stabilization of the droplets without imparting the viscosity of the suspending phase. A narrow polydispersity for polymers is important in colloid science for the preparation of micelles of precise and predictive sizes. However, in our study we use the surfactant to stabilize large nanodroplets. The difference between the molecular weight disperisty (*Đ*_M_ = *M*_w_/*M*_n_) values of the two polymers will therefore not significantly impact the colloidal stabilization.

## 4. Discussion

### 4.1. Stabilization of Inverse Miniemulsions by PTIPSA

The higher molecular weight polymer (10,100 g·mol^−1^) was used to stabilize inverse (water-in-cyclohexane) miniemulsions, because it showed better stability than PTIPSA with 4600 g·mol^−1^. Therefore, water-in-oil miniemulsions were prepared with a concentration of 9 mg·mL^−1^ PTIPSA in cyclohexane. However, no stable emulsion was achieved. Thus, we selected water-free formamide as polar solvent to replace water, because the interfacial tension between cyclohexane and formamide (γ = 21.6 mN·m^−1^ at 22 °C, Figure S4) is lower than the one of cyclohexane and water (γ = 48.7 mN·m^−1^ at 22 °C, Figure S4, literature γ = 50.2 mN·m^−1^ at 20 °C [49]). Water-free formamide was also chosen to eliminate the possible hydrolysis of the TIPS-group during the nanocapsules synthesis. After testing the surface active properties of PTIPSA in cyclohexane (PTIPSA concentration of 9 mg·mL^−1^) by interfacial tension measurement against formamide (γ = 10.3 mN·M^−1^ at 22 °C, Figure S4), stable droplets of formamide in cyclohexane were formed using the inverse miniemulsion procedure due to the lower interfacial tension of formamide-in-cyclohexane compared to water-in-cyclohexane.

**Figure 2:**
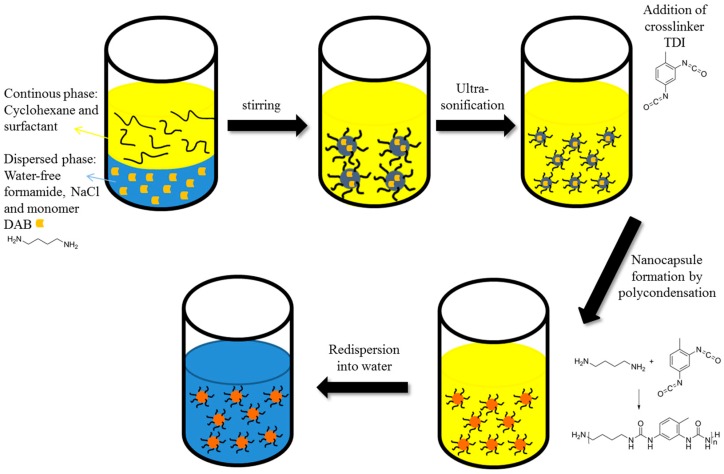
Procedure of an inverse miniemulsion with 1,4-diaminobutane (DAB) and toluene-2,4-diisocyanate (TDI) as monomers to generate polyurea (PU) nanocapsules and their re-dispersion into water.

**Figure 3:**
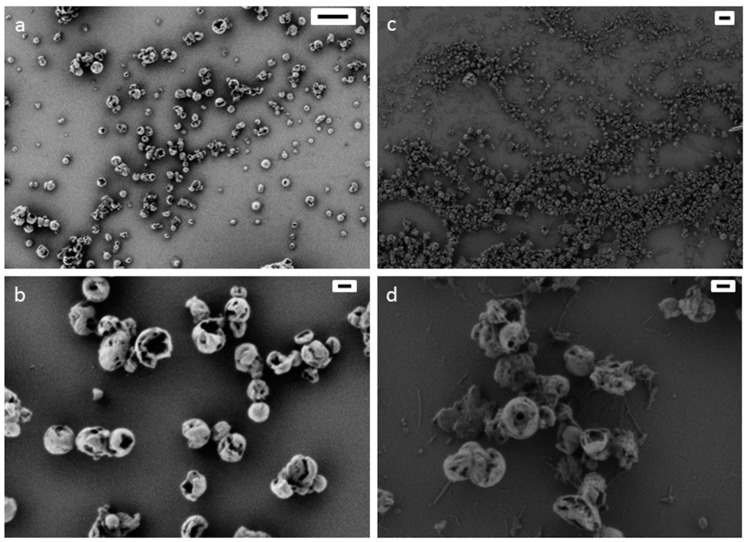
SEM images of polyurea (PU) nanocapsules before ((**a**) scale bar 1 μm; (**b**) scale bar 100 nm) and after redispersion ((**c**) scale bar 1 μm; (**d**) scale bar 100 nm) into 0.1 wt % SDS solution.

To check the utility of such a system for further synthesis of nanoparticles, the droplets of formamide-in-cyclohexane miniemulsions were then used as nanoreactors for the fabrication of polyurea nanocapsules by an interfacial polyaddition reaction (Figure 2). We first verified that the triisopropylsilyl protection groups were stable in the presence of the monomers used in the polyaddition reaction. For this, 1,4-diaminobutane (DAB) or toluene-2,4-diisocyanate (TDI) was added to a PTIPSA solution in cyclohexane-*d_12_* and was analyzed by ^1^H-NMR spectroscopy after stirring for 3 days. In both cases, no cleavage of the protecting group was observed. The monomer DAB and the lipophobe sodium chloride in anhydrous formamide were added to the surfactant solution. After the emulsion was formed, the second monomer TDI was added via the hydrophobic phase to form polyurea nanocapsules by interfacial polycondensation. Whereas the miniemulsions with the lower molecular weight polymer (4600 g·mol^−1^) were not stable or formed agglomerated nanocapsules (*d* > 1 μm), the miniemulsions with the higher molecular weight (10,100 g·mol^−1^) and a surfactant concentration of 9 mg·mL^−1^ yielded capsules with z-average sizes of 306 nm (DLS, Figure S5). After the polyaddition reaction, the typical morphology of hollow nanocapsules was detected by electron microscopy (see Figure 3 and Figure S6). The nanocapsules determined in Figure 3 are collapsed and broken due to high vacuum chamber (3.8 × 10^−6^ mbar) during electron microscopy measurement and led to evaporation of the liquid core independent of the polymer shell material [60,61,62].

### 4.2. Deprotection of the Triisopropylsilyl Groups

For the study of the deprotection of the triisopropylsilyl groups by ^1^H-NMR spectroscopy, the monomer TIPSA was chosen as model molecule because we always observed a phase separation for the investigations of the deprotection of PTIPSA in solution. Indeed, there was no common solvent for the PTIPSA, the hydrolyzed PTIPSA, and the leaving protecting group. TFA was selected for the cleavage of the protecting group because it was used successfully for the *t*-butyldimethylsilyl protection groups [63,64]. Complete deprotection was detected after 1 h for TFA concentrations of 1 M and 0.1 M in the reaction solution (Figure S7). Therefore, the cleavage of the TIPS group is fast although it is anticipated that the deprotection should take longer time for the polymer. Furthermore, partial hydrolysis of the TIPS group was observed in water after 2 days without adding a catalyst or cleavable agent into the solution (Figure S8).

### 4.3. Transfer of the Nanocapsules in Water

The transfer of the nanocapsules from oil to water is crucial for their use in biomedical applications. Both TFA concentrations (1 M and 0.1 M) were prepared and tested in parallel. The TFA solutions were mixed with a defined amount of miniemulsion (1 g in 5 g TFA solution). During the re-dispersion process, the evaporation of cyclohexane in water was found to take around 3 h—as detected by ^1^H-NMR analysis against DMF as an internal standard—meaning that the deprotection should be faster or in the same time frame as for the evaporation of cyclohexane. No stable dispersion was formed after evaporation of cyclohexane, meaning that the deprotected PTIPSA alone is not efficient enough to stabilize the nanocapsules in the aqueous solutions of TFA. Indeed, large agglomerates were generated with diameters larger than 500 nm (Figure S5). The agglomeration was attributed to the acidic environment as the formed polyacrylic acid remained protonated and could therefore not contribute to an electrostatic stabilization of the nanocapsules. Aggregates were still present after neutralization. To overcome this issue, a slight amount of water-soluble surfactant (SDS) was added in the suspending phase (reaching concentrations of 0.1 wt % or 0.01 wt % SDS in the water phase). Indeed, stabilization under acidic conditions with lower SDS concentration was not efficient, because large agglomerates were formed (*d* > 3 μm).

Thus under acidic conditions (1 M TFA, low pH ~ p*K*_a_[TFA]) the addition of only 0.1 wt % or 0.01 wt % SDS were sufficient for the stabilization of the nanocapsule dispersion in water. The nanocapsules stabilized with the lower SDS concentration (0.01 wt % in 1 M TFA, Figure S5) also showed large agglomerates (*d* > 3 μm). The dispersion stabilized with the higher SDS concentration (0.1 wt % in 1M TFA) displayed particles with an average diameter of 203 nm, but also with a small amount of agglomerates (*d* > 4 μm, Figure S5). Under acidic conditions the addition of SDS seems to be necessary to obtain stable nanocapsules. Therefore, we also used only SDS (0.1 wt %) as surfactant for the redispersion step without adding a chemical for deprotection of the silyl group. With 0.1 wt % SDS solution, we could generate stable nanocapsules with a diameter of 406 nm (Figure 3, Figures S5 and S6).

## 5. Conclusions

We demonstrated that poly(acrylic acid) protected with isopropyl silyl groups could stabilize polyurea nanocapsules produced by an inverse miniemulsion process. No amphiphilic block copolymer was needed for the stabilization in the cyclohexane phase. To prepare the stabilizer, isopropylsilyl acrylate was polymerized with 4-cyano-4-(phenylcarbonothioylthio)pentanoic acid as chain transfer agent by the RAFT polymerization process. The polymer was found to stabilize formamide-in-cyclohexane miniemulsions and polyurea nanocapsules were successfully formed. Afterwards the miniemulsion could be re-dispersed with a minimal amount of additional surfactant. This method therefore overcomes the major issue of the presence of the hydrophobic blocks of amphiphilic block copolymer surfactant that are present on the surface of nanocarriers after re-dispersion in water.

## Supplementary Materials

Supplementary Materials can be found at www.mdpi.com/2073-4360/8/8/303/s1.
