# Supplementary material for: Stabilization of Inverse Miniemulsions by Silyl-Protected Homopolymers
Source: Polymers (Basel). 2016 Aug 12;8(8):303. doi: 10.3390/polym8080303 (PMC6431836; doi:10.3390/polym8080303)
Supplement: Supplementary file 1 [file polymers-08-00303-s001.pdf]

Polymers **2016**, *8*, 303; doi:10.3390/polym8080303

Supplementary Materials: Stabilization of Inverse Miniemulsions by Silyl-Protected Homopolymers

Sarah Wald, Frederik R. Wurm, Katharina Landfester and Daniel Crespy

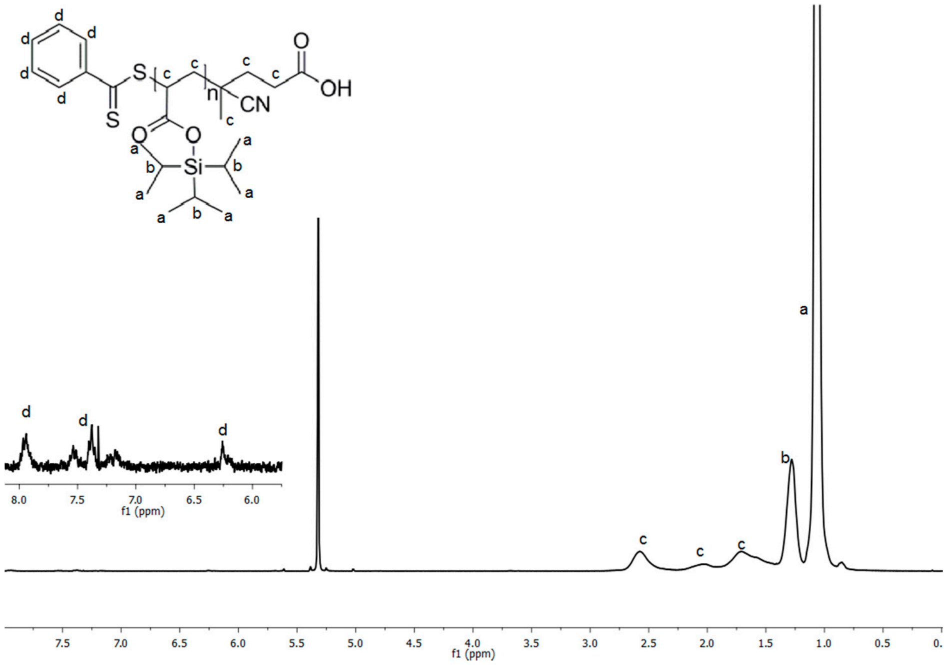

(**a**)

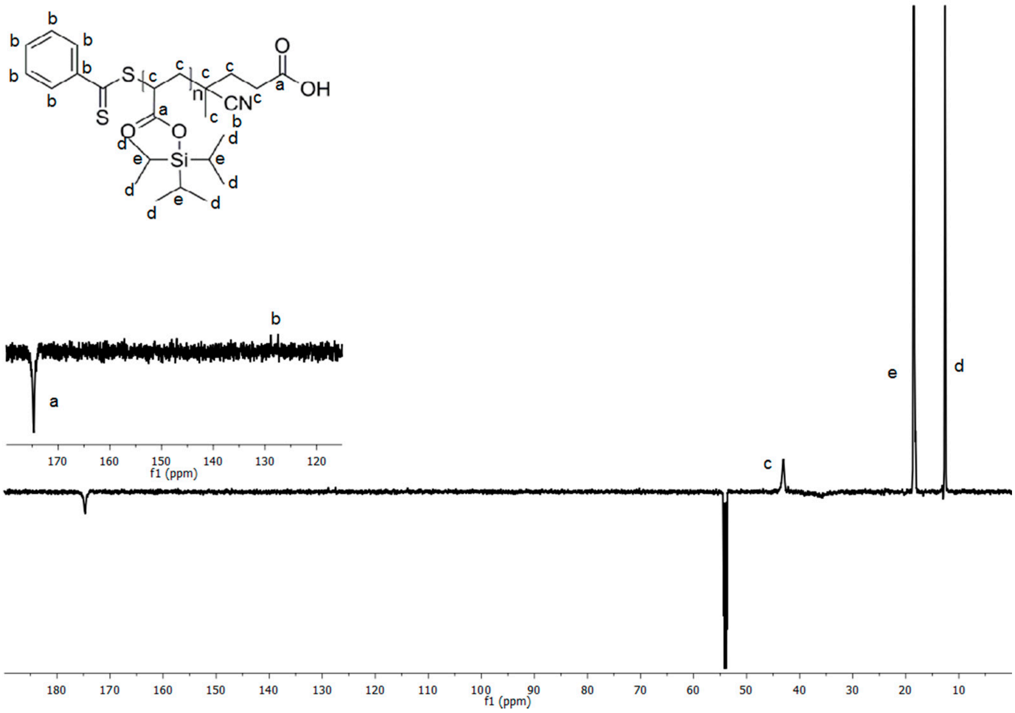

(**b**)

Figure S1. ^{1}H-NMR (**a**) and ^{13}C-NMR (**b**) spectra of poly(triisopropylsilyl acrylate) (PTIPSA) in deuterated dichloromethane.

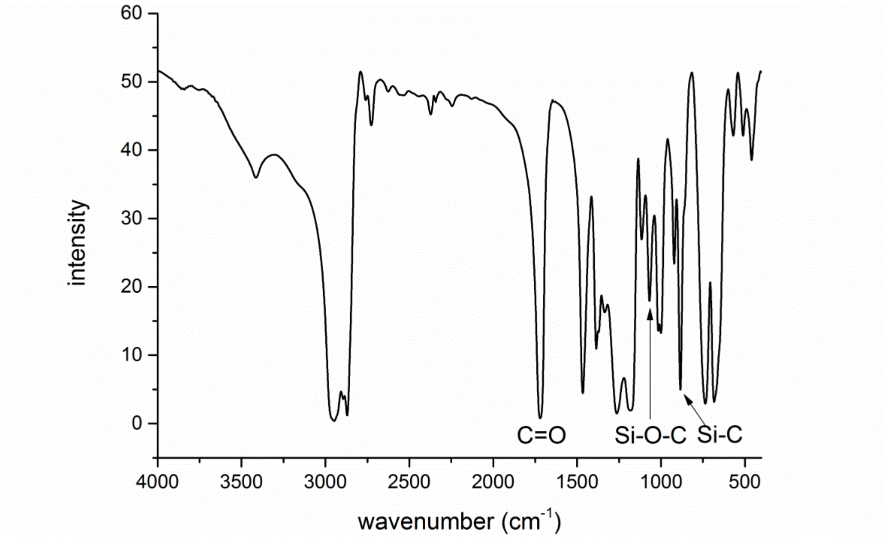

Figure S2. IR spectrum of PTIPSA.

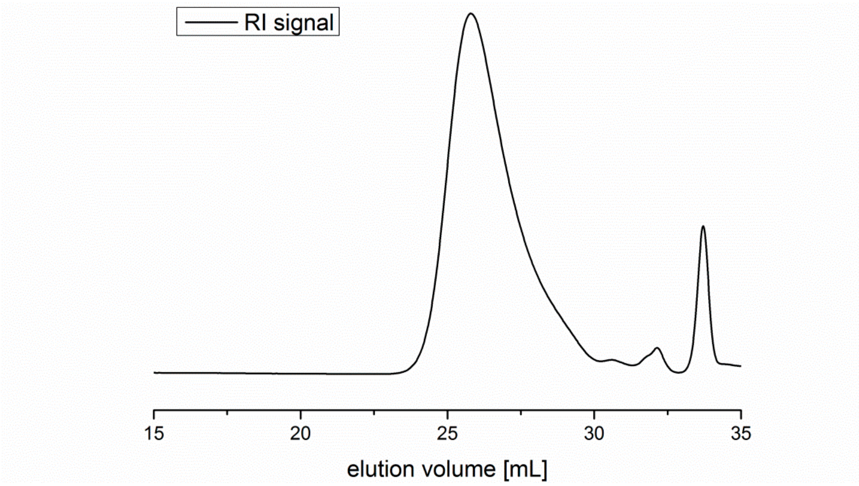

Figure S3. SEC (Size Exclusion Chromatography)trace of PTIPSA (M_n = 10,100 g/mol, M_w = 16,800 g/mol, PDI = 1.67).

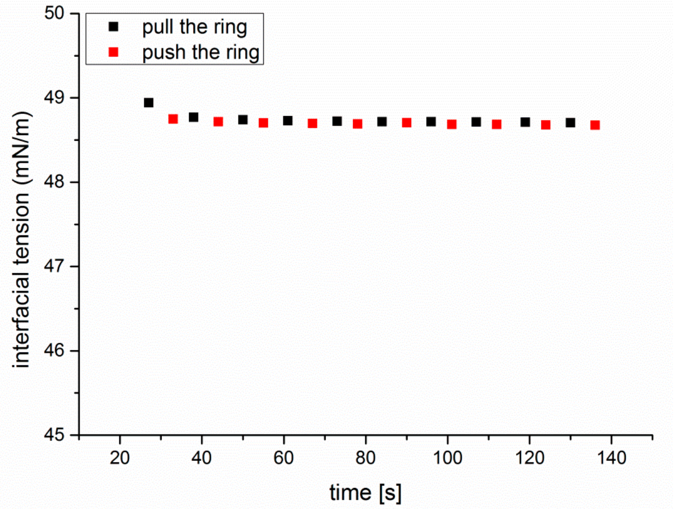

(**a**)

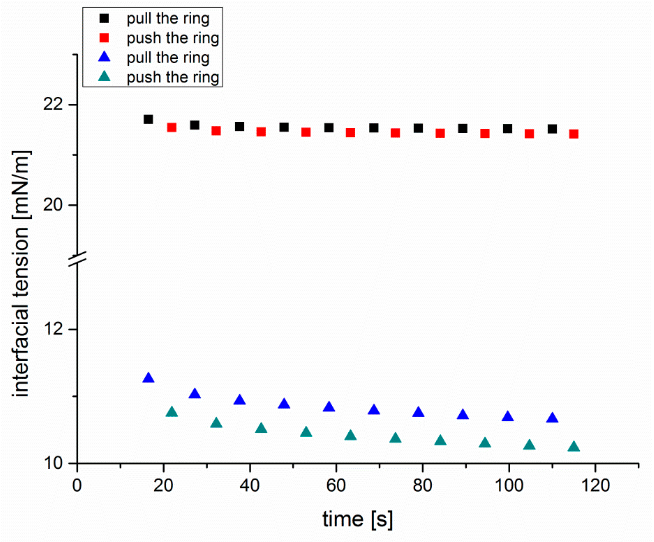

(**b**)

Figure S4. Interfacial tension measurement of cyclohexane and water (σ = 48.7 mN/m at 22 °C) (**a**) and water-free formamide and cyclohexane (σ = 21.6 mN/m at 22 °C) as well as water-free formamide and PTISPA-cyclohexane solution (σ = 10.3 mN/m at 22 °C) (**b**).

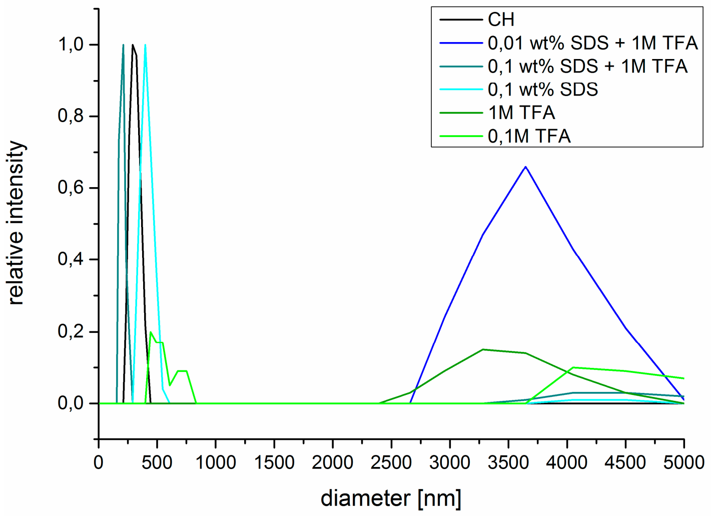

Figure S5. DLS results of the polyurea nanocapsules in cyclohexane and after redispersion into the different water mixtures.

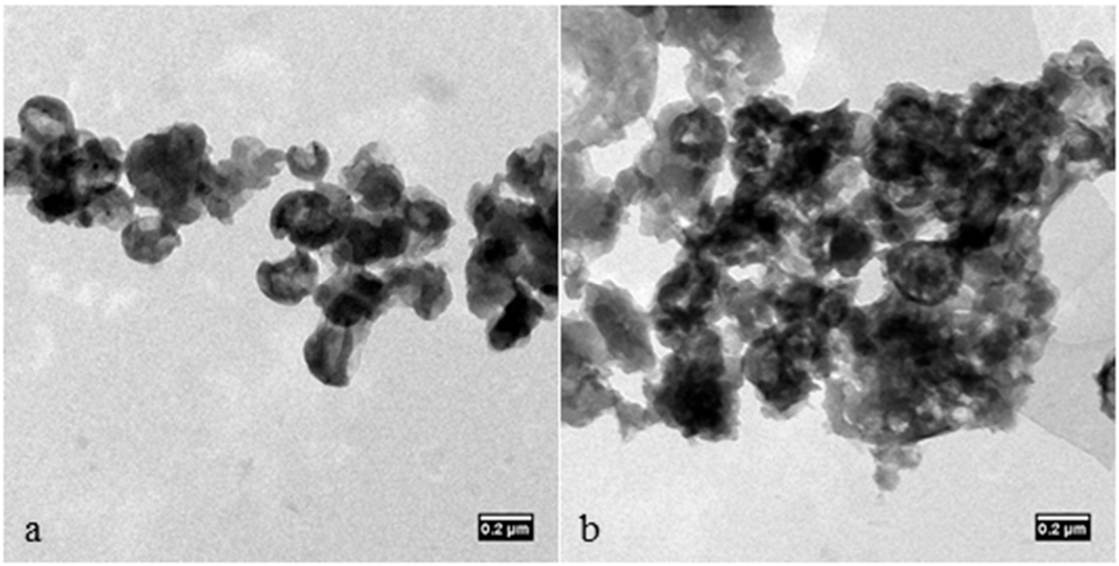

Figure S6. TEM images of polyurea nanocapsules in cyclohexane (**a**) and after redispersion in 0.1 wt % aqueous SDS solution (**b**).

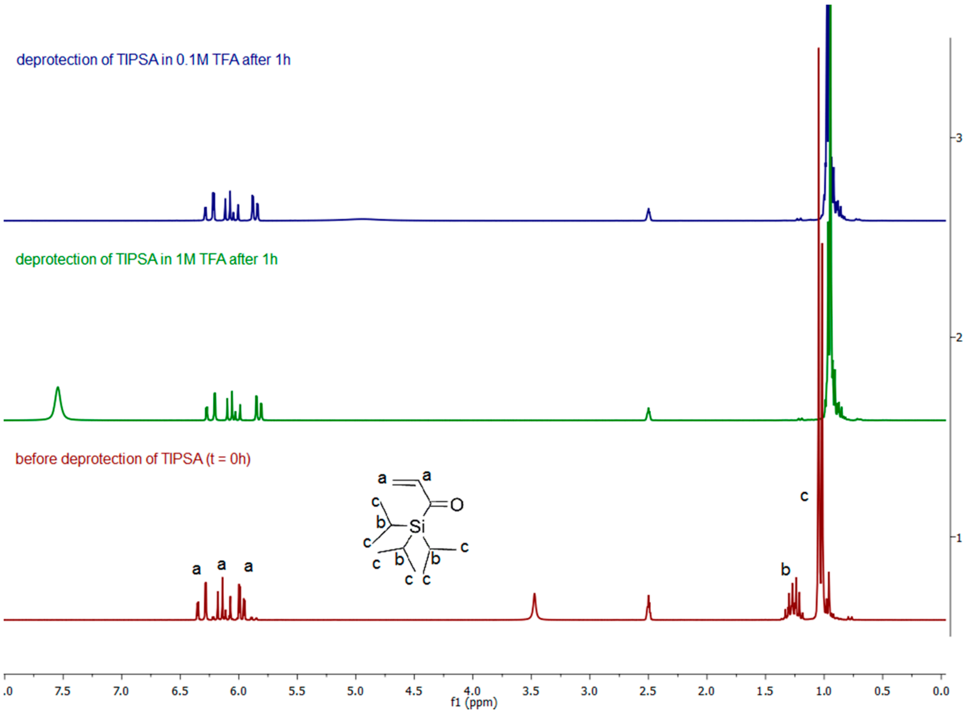

Figure S7. Kinetic measurements of deprotection of TIPSA using TFA measured by ^{1}H-NMR spectroscopy in a solvent mixture of DMSO-d_6 and D_2O (red: t = 0 h, green: deprotection in 1M TFA at t = 1 h, blue: deprotection in 0.1M TFA at t = 1 h). The signal around 1.38–1.16 ppm completely disappeared and the signal at 1.03 shifted form the red to the blue and green spectra in 1.00–0.83 ppm region.

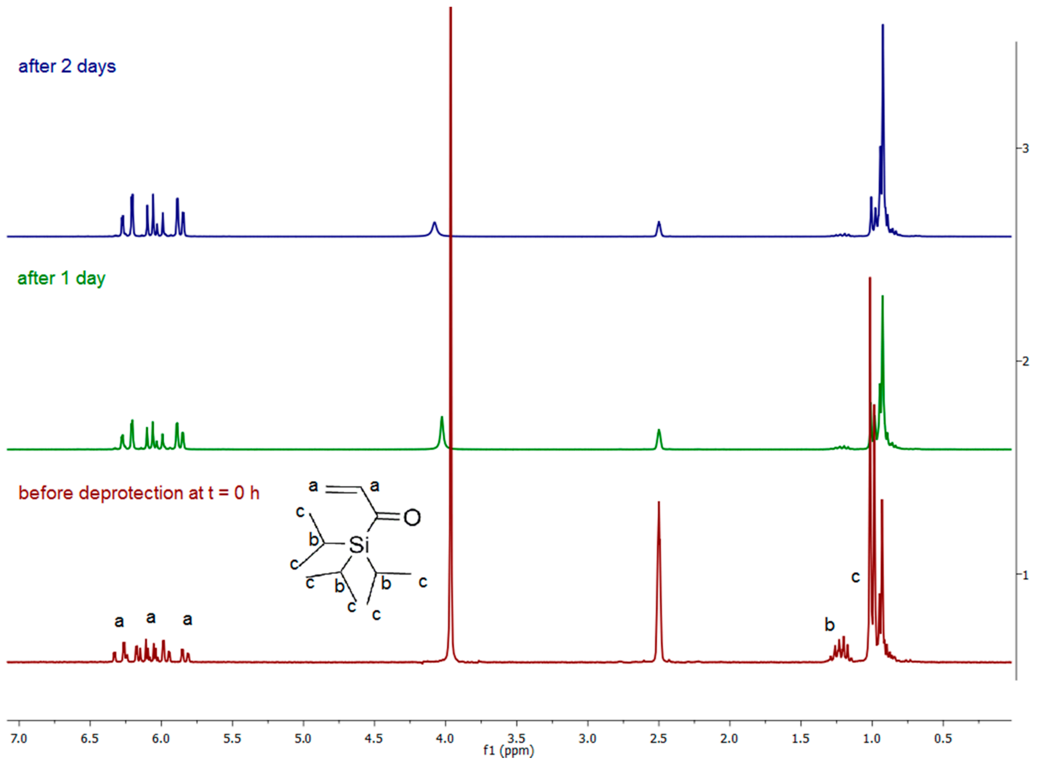

Figure S8. Kinetic measurements of deprotection of TIPSA with water measured by ^{1}H-NMR spectroscopy in a solvent mixture of DMSO-d_6 and D_2O (red: *t* = 0 h, green: *t* = 1 day, blue: *t* = 2 days). The signal around 1.38–1.16 ppm completely disappeared and the signal at 1.03 shifted from the red to the blue and green spectra in 1.00–0.83 ppm region.

© 2016 by the authors; licensee MDPI, Basel, Switzerland. This article is an open access article distributed under the terms and conditions of the Creative Commons Attribution (CC-BY) license (http://creativecommons.org/licenses/by/4.0/).
